# Vitamin D and Human Health: Celebrating Diversity

**DOI:** 10.3390/nu6010011

**Published:** 2013-12-19

**Authors:** Simon Spedding

**Affiliations:** Nutritional Physiology Research Centre, University of South Australia, Adelaide, South Australia 5000, Australia; E-Mail: spedding@adam.com.au; Tel.: +61-439-687-886

This Special Issue of *Nutrients*: *Vitamin D and Human Health* celebrates diversity in vitamin D research with articles from bench-to-bedside, examining mechanisms, epidemiology, and clinical issues in the management of non-skeletal disease following themes set by an earlier review in *Nutrients* [1]. Vitamin D became synonymous with calcium and bone metabolism originating from Casimir Funk’s concept of “Vitamines”. This suggests that vitamin D is an amine found in food with a single mode of action affecting calcium and bone metabolism [2], whereas vitamin D is a secosteroid hormone derived from sunshine with a plethora of physiological functions (autocrine, paracrine, endocrine [3], and epigenetic [4]) associating vitamin D deficiency with many illnesses [1]. Deficiency is pandemic and most prevalent where sun exposure is limited by culture climate and skin colour [5]. Whilst reports have focused on diet and bone metabolism [6], this Special Issue of *Nutrients* about *Vitamin D and Human Health* focuses on non-skeletal disease, and research driven by industry and community health concerns.

Community concerns drive some vitamin D research. Whilst Canadians have inadequate vitamin D intakes [7], Vatanparast and colleagues [8] found migrant and refugee children in Canada had even lower intakes and lower 25(OH)D levels such that refugee children had half the intake of Canadian children, indicating that migration is a significant risk factor for vitamin D deficiency. When the daily intake of vitamin D was equivalent to 400–800 IU as suggested by the Institute of Medicine [6], Chao and colleagues [9] found that 25(OH)D levels did not improve. When managing deficiency, the authors believe significantly higher doses are required and individual factors should be considered. The high prevalence of deficiency during pregnancy across Asia led Roth and colleagues [10] to examine the dose response and tolerability of high dose vitamin D supplementation in Bangladesh. Sufficiency (>80 nmol/L) was safely attained for most pregnant women with 35,000 IU a week, however, only half of the pregnant women attained sufficiency when taking a lower dose of 14,000 IU a week. Another factor that influences 25(OH)D is hormonal contraception, Moller and colleagues [11] found 13%–25% higher concentrations of vitamin D and its binding protein. Community attitudes to vitamin D and sun exposure were examined by Bonevski and colleagues [12], finding that Australians at high risk of vitamin D deficiency perceived themselves to be more at risk of skin cancer than deficiency. The authors advocate for clearer public health messages about balanced sun-exposure.

Industry also drives vitamin D research. DSM^©^ focused on the evidence-base for the use of vitamin D supplements by supporting a meta-analysis by Dong and colleagues [13] showing vitamin D intake is associated with a 30% reduced risk of developing Type 1 diabetes when taken during infancy. Roche sponsored a study by Abdel-Wareth and colleagues [14] to compare diagnostic systems accuracy of testing for vitamin D to HPLC for measuring 25(OH)D levels found good concordance in all samples except those with high serum D2 (>10 nmol/L).

The broad health impact of vitamin D was reviewed by Spedding and colleagues [15]. Levels of evidence for the management of diseases with vitamin D include level 1 evidence for premature mortality and falls prevention from meta-analyses, and level 2 evidence for cancer, diabetes, pain, depression and respiratory infections from randomised controlled trials (RCTs). Furthermore the authors present evidence to support the hypothesis that different levels of 25(OH)D levels are required for different diseases and specifically higher levels are required for non-skeletal diseases such as all-cause mortality, falls and cancer than for bone disease.

Cancer prevention was reviewed by Moukayed and Grant [16]. The immediate nongenomic responses and persistent genomic effects of vitamin D involved in regulating cell proliferation differentiation and survival. This is consistent with ecological and epidemiological studies suggesting vitamin D reduces the risk of cancer. This is supported by two RCTs. Whilst researchers rely on finding gaps, clinicians and policy makers need to base advice on the best evidence available, thus the authors recommend countries adopt awareness, education, and implementation strategies to increase supplementation with vitamin D as a preventive measure to reduce colon and breast cancer.

For cardiometabolic disease, effects on blood pressure and vitamin D were found to be dose dependant by Liu and colleagues [17] and doses of 800 IU/day were more effective than 400 IU/day. Vanlint [18] describes conflicting outcomes of obesity studies that may reflect flaws in the studies rather than the effect of vitamin D. Hall and colleagues [19] suggest that in haematopoiesis vitamin D may have a positive role through immune modulation. Two papers explored Pagani’s theory of a sympathovagal balance (SVB) and vitamin D deficiency. Mann and colleagues [20] demonstrated SVB suppression in vitamin D deficiency and postulated that deficiency increased cardiovascular risk. The cardioprotective effect of Vitamin D supplementation through suppression of sympathovagal balance was studied in cardiocytes by Pacini and colleagues [21].

For athletes, muscle weakness after exercise correlated with low levels of 25(OH)D, therefore Barker and colleagues [22] postulate that vitamin D influences recovery of muscle strength. Ogan and colleagues [23] found that Vitamin D deficiency is common in athletes especially indoor athletes and one study indicated that performance improved when 25(OH)D was above 100 nmol/L. This may have importance for athletic performance and possibly for falls prevention in the elderly.

In conclusion, this Special Issue of *Nutrients:* *Vitamin D and Human Health* expands the literature on vitamin D by exploring mechanisms epidemiology and trials that demonstrate the broad health effects of vitamin D deficiency and the influence of consumers and industry on research. The “accepted wisdom” that vitamin D should be limited to bone disease is based on evidence from research that used insufficient doses of Vitamin D and from RCTs that considered vitamin D as a drug, ignoring its particular mechanisms and the other sources of vitamin D [16]. This evidence also supports the contention that just as vitamin D metabolism is tissue dependent, so vitamin D sufficiency is a disease dependent concept [15]. The lessons learnt from vitamin D research may apply across the spectrum of nutrients and the future of nutrient research without these limitations in study design may usher in a new age in their understanding.
